# Evaluation of Contrast Sensitivity after Single Intravitreal Triamcinolone Injection for Macular Edema Secondary to Branch Retinal Vein Occlusion

**DOI:** 10.1155/2013/549240

**Published:** 2013-10-03

**Authors:** Tulin Aras Ogreden, Zeynep Alkin, Abdullah Ozkaya, Halil Ibrahim Demirkale, Irfan Perente, Cengiz Aras

**Affiliations:** ^1^Suleymaniye Women Health Hospital, Istanbul, Turkey; ^2^Beyoglu Eye Training and Research Hospital, Bereketzade Cami Sok No. 2, Beyoglu, Istanbul, Turkey; ^3^Dunya Goz Hospital, Istanbul, Turkey

## Abstract

*Purpose.* To evaluate visual acuity (VA), contrast sensitivity (CS), and central retinal thickness (CRT) after intravitreal triamcinolone acetonide (IVT) injection for macular edema secondary to branch retinal vein occlusion (BRVO). *Methods.* In this prospective study, a total of 21 eyes of 21 patients were included. VA, CS, and CRT were assessed at baseline and at 1, 3, and 6 months after a single IVT injection. *Results.* Mean age was 64.57 ± 8.34 years. The mean baseline VA (LogMAR) increased from 1.11 ± 0.63 to 0.55 ± 0.39 (*P* < 0.001), 0.60 ± 0.40 (*P* < 0.001), and 0.78 ± 0.39 (*P* = 0.07) at 1, 3, and 6 months, respectively. The mean baseline CS (log CS) at 1 meter improved from 0.66 ± 0.49 to 1.11 ± 0.32 (*P* < 0.001), 0.99 ± 0.38 (*P* < 0.001), and 0.72 ± 0.37 (*P* = 0.8) at 1, 3, and 6 months, respectively. The mean baseline CS (log CS) at 3 meters improved from 0.34 ± 0.41 to 0.74 ± 0.41 (*P* < 0.001), 0.64 ± 0.44 (*P* = 0.036), and 0.46 ± 0.49 (*P* = 0.8) at 1, 3, and 6 months, respectively. The mean baseline CRT decreased from 511 ± 146 *μ*m to 242 ± 119 *μ*m, 277 ± 131 *μ*m, and 402 ± 166 *μ*m at 1, 3, and 6 months after IVT (*P* < 0.001 for each). *Conclusion.* Single IVT injection improved VA and CS and reduced CRT at 1 and 3 months of treatment. VA and CS returned to baseline levels at 6 months.

## 1. Introduction

Macular edema (ME) is the most frequent complication of branch retinal vein occlusion (BRVO) [1]. Since the Branch Vein Occlusion Study Group reported efficacy of grid laser photocoagulation, it has been accepted as the standard treatment for ME secondary to BRVO [2]. Recently, an increasing number of reports have revealed the efficacy of new treatment options for ME secondary to BRVO, such as intravitreal injection of triamcinolone acetonide (IVT), bevacizumab, ranibizumab, or aflibercept [3–7]. As various therapies are currently available, more detailed clinical assessment of the visual functions of these patients has become more important. Most of the previous studies evaluating the outcomes of intravitreal agents in eyes with ME secondary to BRVO assessed visual function with VA measurement [3–6]. VA is one of the components of functional vision and measures standard high contrast visual acuity which is not a true reflection of visual performance [8]. VA is associated with tasks requiring good resolution and adaptation to changing light conditions, whereas contrast sensitivity (CS) is associated with daily activities requiring distance judgements, night driving, and mobility [9]. Thus, CS function may give an additional information on visual performance for evaluating functional results of such treatments. 

Therefore, the purpose of the present study is to evaluate prospectively the effect of a single IVT injection on retinal anatomy and functions as reflected by CRT, VA, and CS testing in patients with ME secondary to BRVO followed up for 6 months.

## 2. Materials and Methods

This prospective study consisted of 21 patients who had ME secondary to unilateral BRVO and were treated with IVT between February 2006 and October 2007. The procedures used in this study adhered to the tenets of the Declaration of Helsinki, and written informed consent was obtained from each patient after the nature and possible consequences of the study were explained.

The inclusion criteria were eyes with treatment naive BRVO, central retinal thickness (CRT) greater than 250 *μ*m involving the foveal center, and visual acuity equal to or less than 20/40. The patients who had prior ocular surgery, macular laser photocoagulation, and intravitreal treatment with anti-VEGF agents/IVT or had coexisting retinal disease such as diabetic retinopathy, epiretinal membrane, or media opacities that could decrease visual acuity, or macular ischemia were not included. 

All patients underwent a complete ophthalmic examination, including best-corrected VA measurement with Early Treatment Diabetic Retinopathy Study chart, slit-lamp biomicroscopy, indirect fundus ophthalmoscopy, OCT imaging using OCT 2000 scanner (Humphrey Instruments, San Leandro, CA, USA), fluorescein angiography using Heidelberg Retinal Angiograph (Heidelberg Retina Angiography; Heidelberg Engineering, Heidelberg, Germany), and CS testing at baseline and at 1, 3, and 6 months. 

Contrast sensitivity at 1 and 3 meters with the best distance correction was evaluated using the Pelli-Robson test (Lombart Instrument, USA) in the same photopic conditions (~80 candelas/m^2^). The results were recorded as the line with the majority of correctly identified letters. 

CRT was defined as the distance between internal limiting membrane and the retinal pigment epithelium (RPE) at the center of the fovea.

All patients were treated with a single intravitreal injection of 4 mg/0.1 mL triamcinolone (Kenacort-A; 40 mg/mL; Bristol-Myers Squibb Co, Princeton, NJ, USA) within a week after the admission. After instillation of topical 0.05% proparacaine hydrochloride, the periocular skin was cleaned with 10% povidone iodine. Then the eye to be injected was draped with an eye towel and plastic adhesive drape, the conjunctival sac was irrigated with 5% povidone iodine, and the eye was opened using a lid retractor. The drug was injected through the pars plana, 3.5 mm posterior to the limbus, using a 27-gauge needle on a 1 mL syringe. After the intravitreal injections, an ophthalmic solution of topical levofloxacin was administered 5 times a day for a week. Patients were examined on days 1 and 7 to detect any sign of infection.

Statistical analysis of the data was carried out with SPSS software (SPSS v 16.0; Inc., Chicago, IL, USA). Data regarding VA was converted to logarithm of the minimum angle of resolution (Log MAR) before analysis and calculations. All values are presented as mean with standard deviation. A one-way ANOVA with repeated measures was used to compare baseline CS, VA, and OCT parameters with the values obtained at 1, 3, and 6 months. The two-sided significance level was set at *P* < 0.05.

## 3. Results

A total of 21 eyes of 21 patients (13 women and 8 men) were evaluated. The mean age of the patients was 64.57 ± 8.34 years (range: 52–79 years). The mean duration of symptoms was 5.4 ± 4.7 months (range: 3–12 months). The right eye was involved in 9 patients (42.8%) and the left eye in 12 (57.2%). Thirteen (61.9%) of the patients presented superotemporal BRVO, and 8 (38.1%) of the patients presented inferotemporal BRVO. 

The mean baseline VA (log MAR) was 1.11 ± 0.63 (range: 2.31–0.11) and increased significantly to 0.55 ± 0.39 at 1 month (*P* < 0.001) and 0.60 ± 0.40 at 3 months (*P* < 0.001). The mean VA was 0.78 ± 0.39 Log MAR at 6 months, and the change was not statistically significant compared to baseline (*P* = 0.07).

**Figure 1:**
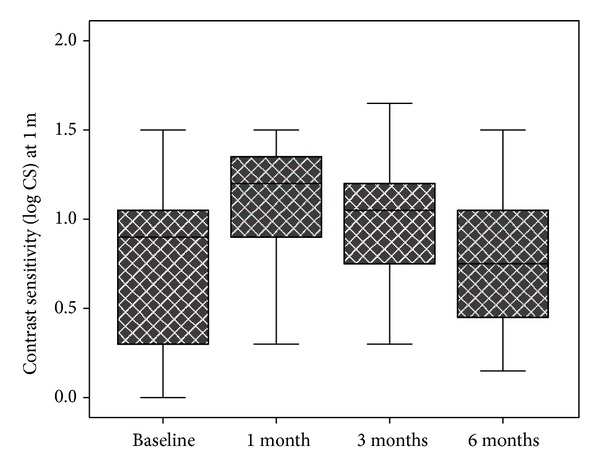
Box plot chart showing contrast sensitivity at 1 meter before and 1 month, 3 months, and 6 months after intravitreal triamcinolone injection in eyes with macular edema secondary to branch retinal vein occlusion.

The mean baseline CS (log CS) at 1 meter was 0.66 ± 0.49 (range: 0.00–2.25) and improved significantly to 1.11 ± 0.32 at 1 month (*P* < 0.001) and 0.99 ± 0.38 at 3 months (*P* < 0.001). Mean CS (log CS) was 0.72 ± 0.37 at 6 months and the change was not statistically significant compared to baseline (*P* = 0.8).  Figure 1 shows the CS changes at 1 meter during the follow-up period. 

**Figure 2:**
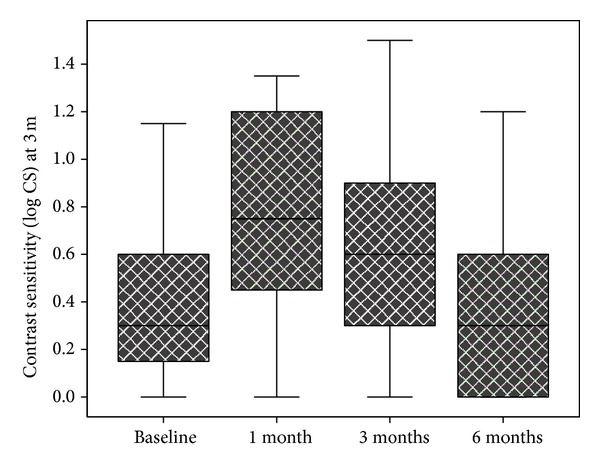
Box plot chart showing contrast sensitivity at 3 meters before and 1 month, 3 months, and 6 months after intravitreal triamcinolone injection in eyes with macular edema secondary to branch retinal vein occlusion.

The mean baseline CS (log CS) at 3 meters was 0.34 ± 0.41 (range: 0.00–1.15) and improved significantly to 0.74 ± 0.41 at 1 month (*P* < 0.001) and 0.64 ± 0.44 at 3 months (*P* = 0.036). Mean CS (log CS) was 0.46 ± 0.49 at 6 months and the change was not statistically significant compared to baseline (*P* = 0.6).  Figure 2 shows the CS changes at 3 meters during the follow-up period. 

The mean baseline CRT was 511 ± 146 *μ*m (range: 264 *μ*m–740 *μ*m) and decreased significantly to 242 ± 119 *μ*m at 1 month, 277 ± 131 *μ*m at 3 months, and 402 ± 166 *μ*m at 6 months (*P* < 0.001 for each comparison). 

Four (19%) of the 21 patients showed an increase of IOP more than 21 mm Hg during the 6-month followup, but the IOP regulation could be achieved with medical therapy in all of the patients. 

## 4. Discussion

Macular edema is the most frequent complication of BRVO and may cause severe visual impairment in some of the patients [1]. Intravitreal corticosteroids have been used for the treatment of ME in a great number of studies because of their ability to inhibit the arachidonic acid pathway and downregulate the production of vascular endothelial growth factor [3–5]. Triamcinolone also reestablishes the blood-retinal barrier by modulating the expression of intracellular adhesion molecule 1. In addition, triamcinolone has the potential to influence cellular permeability, including the barrier function of the retinal pigment epithelium [10]. In previous studies, intravitreal triamcinolone had been successfully used for the treatment of ME secondary to BRVO [3–5]. 

The SCORE study which was a multicenter, randomized clinical trial compared the efficacy and safety of standard care versus IVT in the treatment of ME in patients secondary to BRVO [11]. An increase in VA score of 15 or more letters from baseline to 12 months was achieved in 29%, 26%, and 27% of the patients in the standard care and 1 and 4 mg IVT groups, respectively. No difference was found in VA at 12 months among the groups. Nowadays, IVT is used less frequently because of complications such as increase of IOP and progression, or formation of cataract, and most patients receive anti-VEGF treatment instead. Our study results were collected when IVT was more popular in the treatment of ME secondary to BRVO. 

In previous studies evaluating the safety and efficacy of intravitreal steroids, VA was a standard way to measure the visual function [3–5]. However, we know that VA measurement poorly describes the functional impact on visual performance in patients with compromised central visual field. Objects in the visual field exhibit varying degrees of contrast and a varying content of spatial frequencies. CS is more closely associated with tasks requiring distance judgment, night driving, and mobility than VA [12]. Considering both VA and CS when assessing the outcomes of clinical trials may provide a more complete picture of the effects of treatment on vision than either measure alone.

Recently, Preti et al. evaluated the change in CS function after intravitreal injection of bevacizumab for the treatment of ME secondary to BRVO [13]. They found that eyes with ME showed an improvement in VA 1 month after bevacizumab injection, but VA again worsened at 3 months. CS test demonstrated a significant improvement at spatial frequencies of 3, 6, 12, and 18 cycles/degree 1 month after injection and at the spatial frequency of 12 cycles/degree at 3 months.

Our results were comparable with the Preti's study. With respect to OCT measured CRT, our results showed a significant decrease from baseline to 1, 3, and 6 months. The greatest decrease in CRT from baseline was observed at 1 month, and this effect was maintained up to 6 months in most of the patients. The results of this prospective study also showed that, after IVT injection, a significant increase in VA improvement up to 3 months was obtained, besides significant decrease in CRT. This effect in VA decreased with time and diminished to insignificant levels at month 6. Many studies evaluating IVT for BRVO have reported a rapid but temporary improvement in VA, which has often required additional injections to maintain the improvement. Similar to VA, CS showed a significant course in improvement during follow-up period after a single injection of IVT. The limitations of our study were a relatively small number of patients, lack of control group, and short follow-up period. 

In our knowledge, our study is the first study that used simultaneous VA measurement, OCT, and CS testing to examine the anatomical and functional changes after IVT for the treatment of ME secondary to BRVO (from a PubMed and MEDLINE search). This study indicated that a single IVT injection produces significant improvement in VA, CS, and CRT measurements up to 3 months, and the improvement regarding CRT measurements lasts for up to 6 months. The diminishing effect of IVT signifies the need for interventions with longer followup. In addition to measurement of VA, testing of CS would be helpful for the evaluation of the effectiveness of IVT in eyes with BRVO. 

## References

[1] McIntosh RL, Mohamed Q, Saw SM, Wong TY (2007). Interventions for branch retinal vein occlusion: an evidence-based systematic review. *Ophthalmology*.

[2] The Branch Vein Occlusion Study Group (1984). Argon laser photocoagulation for macular edema in branch vein occlusion. *American Journal of Ophthalmology*.

[3] Çekiç O, Chang S, Tseng JJ (2005). Intravitreal triamcinolone treatment for macular edema associated with central retinal vein occlusion and hemiretinal vein occlusion. *Retina*.

[4] Cakir M, Dogan M, Bayraktar Z (2008). Efficacy of intravitreal triamcinolone for the treatment of macular edema secondary to branch retinal vein occlusion in eyes with or without grid laser photocoagulation. *Retina*.

[5] Özkiriş A, Evereklioglu C, Erkiliç K, Ilhan Ö (2005). The efficacy of intravitreal triamcinolone acetonide on macular edema in branch retinal vein occlusion. *European Journal of Ophthalmology*.

[6] Campochiaro PA, Heier JS, Feiner L (2010). Ranibizumab for macular edema following branch retinal vein occlusion: six-month primary end point results of a phase III study. *Ophthalmology*.

[7] Campochiaro PA (2012). Anti-vascular endothelial growth factor treatment for retinal vein occlusions. *Ophthalmologica*.

[8] Arden GB (1978). The importance of measuring contrast sensitivity in cases of visual disturbance. *British Journal of Ophthalmology*.

[9] Rubin GS, Bandeen-Roche K, Huang GH (2001). The association of multiple visual impairments with self-reported visual disability: SEE project. *Investigative Ophthalmology and Visual Science*.

[10] Floman N, Zor U (1977). Mechanism of steroid action in ocular inflammation: inhibition of prostaglandin production. *Investigative Ophthalmology & Visual Science*.

[11] Scott IU, Ip MS, VanVeldhuisen PC (2009). A randomized trial comparing the efficacy and safety of intravitreal triamcinolone with standard care to treat vision loss associated with macular edema secondary to branch retinal vein occlusion: the standard care versus corticosteroid for retinal vein occlusion (SCORE) study report 6. *Archives of Ophthalmology*.

[12] Haymes SA, Roberts KF, Cruess AF (2006). The letter contrast sensitivity test: clinical evaluation of a new design. *Investigative Ophthalmology and Visual Science*.

[13] Preti RC, Ramirez LM, Pimentel SL (2012). Single intravitreal bevacizumab injection effects on contrast sensitivity in macular edema from branch retinal vein occlusion. *Arquivos Brasileiros de Oftalmologia*.

